# Operon Gene Order Is Optimized for Ordered Protein Complex Assembly

**DOI:** 10.1016/j.celrep.2015.12.085

**Published:** 2016-01-21

**Authors:** Jonathan N. Wells, L. Therese Bergendahl, Joseph A. Marsh

**Affiliations:** 1 MRC Human Genetics Unit, Institute of Genetics and Molecular Medicine, University of Edinburgh, Western General Hospital, Edinburgh EH4 2XU, United Kingdom

## Abstract

The assembly of heteromeric protein complexes is an inherently stochastic process in which multiple genes are expressed separately into proteins, which must then somehow find each other within the cell. Here, we considered one of the ways by which prokaryotic organisms have attempted to maximize the efficiency of protein complex assembly: the organization of subunit-encoding genes into operons. Using structure-based assembly predictions, we show that operon gene order has been optimized to match the order in which protein subunits assemble. Exceptions to this are almost entirely highly expressed proteins for which assembly is less stochastic and for which precisely ordered translation offers less benefit. Overall, these results show that ordered protein complex assembly pathways are of significant biological importance and represent a major evolutionary constraint on operon gene organization.

## Highlights

• Operon-encoded subunits tend to be encoded by neighboring genes and form large interfaces
• Operon gene order is often optimized for the order of protein complex assembly
• Exceptions are mostly highly expressed proteins for which assembly is less stochastic

## Graphical Abstract

**Figure:**
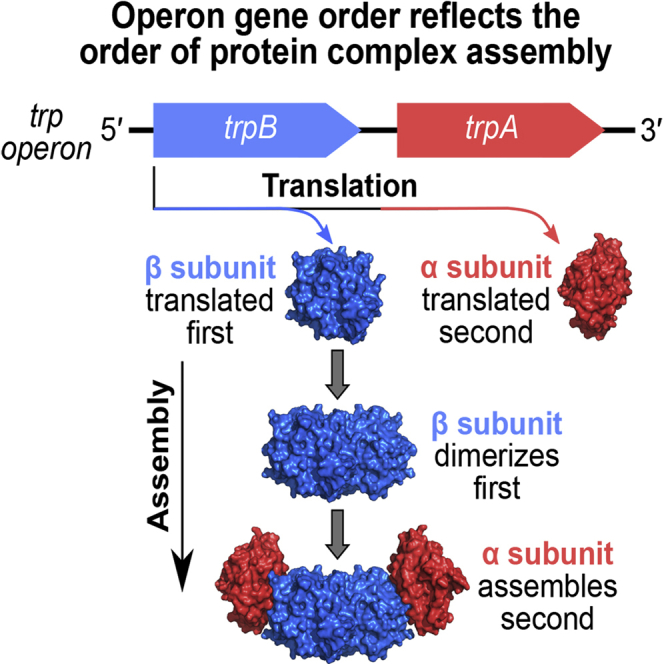

## Teaser

Many prokaryotic protein complexes are operon-encoded, so that subunits of the same complex will be translated from the same mRNA. Wells et al. show that the order in which genes are arranged in operons tends to be optimized for the order in which protein complex subunits assemble.

## Introduction

The assembly of proteins into complexes is integral to a wide range of biological processes. Although we now have extensive knowledge of the diverse quaternary structures formed by protein complexes (Goodsell and Olson, 2000, Janin et al., 2008, Marsh and Teichmann, 2015, Ahnert et al., 2015), much less is known about how they assemble and how assembly is regulated. In recent years, advances in electrospray mass spectrometry techniques have provided major new insights into in vitro assembly, allowing the assembly and disassembly pathways of protein complexes with diverse quaternary structure topologies to be elucidated in detail (Hernández and Robinson, 2007). In homomers, formed from the self-assembly of a single type of polypeptide chain, experimentally identified assembly intermediates often correspond to putative evolutionary precursors, so that the evolutionary history of a complex is reflected in its assembly pathway (Levy et al., 2008). Heteromers, formed from multiple distinct subunits, also tend to assemble and disassemble via ordered pathways that have a strong tendency to be evolutionarily conserved (Marsh et al., 2013). Although these experiments can be time-consuming, ordered assembly pathways can usually be predicted with very good accuracy from the known three-dimensional structure of a complex (Levy et al., 2008, Marsh et al., 2013). Given the many thousands of protein complex structures that are now available, this enables the study of assembly on a larger scale using computationally predicted assembly pathways.

Within the cell, assembly is much more complex and stochastic than in vitro, particularly in heteromers where multiple protein-coding genes must first be transcribed to mRNA and translated into protein, and those proteins must then find each other and assemble. Assembly is especially difficult for lowly expressed proteins, for which the stochastic variations in relative subunit concentrations are greater and the probability of interaction is lower (Kovács et al., 2009, Swain et al., 2002). How do cells cope with this? Does assembly within the cell follow similar ordered pathways as those observed in vitro and predicted computationally? Where does assembly occur within the cell? Has the regulation of gene expression been optimized for protein complex assembly order, as appears to be the case for the large multi-subunit bacterial flagella (Kalir et al., 2001)? Here we were able to address all of these questions by considering the relationship between protein complex assembly and gene organization in prokaryotic operons.

## Results

### Operon-Encoding of Protein Complexes Is Likely to Enhance the Efficiency of Assembly

**Figure 1:**
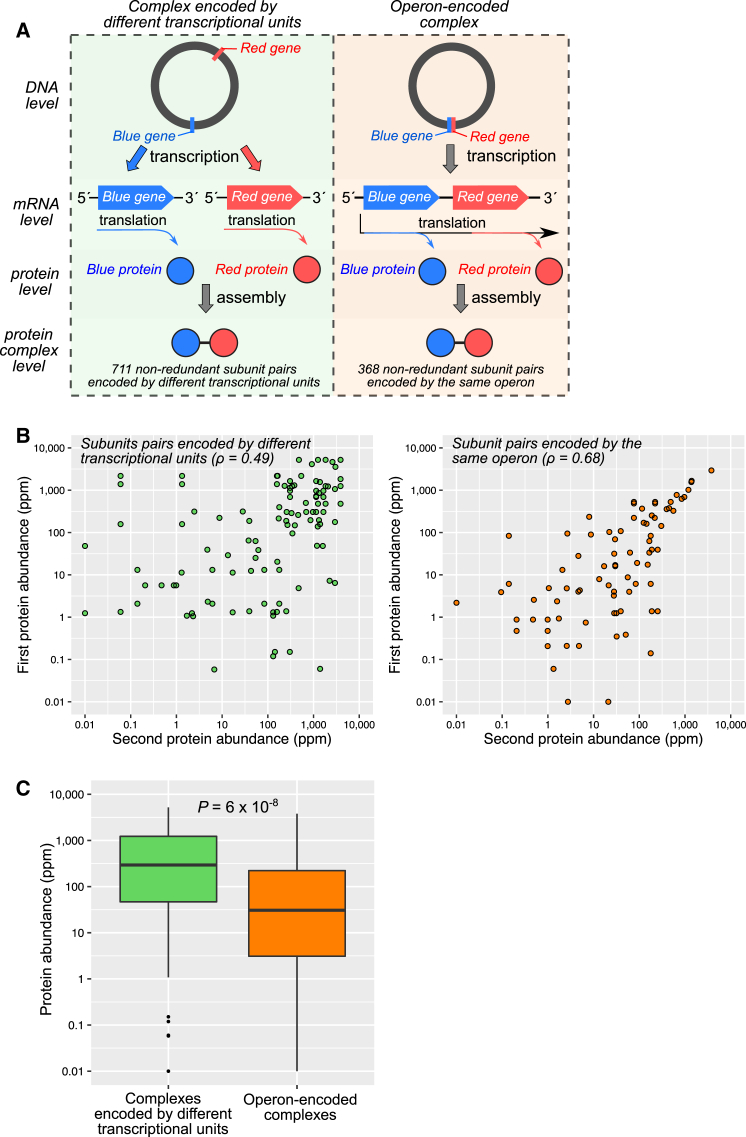
Operon Encoding of Protein Complex Subunits Enhances the Efficiency of Assembly (A) Comparison of assembly for heterodimers where different subunits are encoded by different transcriptional units and where genes encoding both subunits are present on the same operon. (B) Correlation (Spearman’s *ρ*) between abundance measurements from subunit pairs encoded by different transcriptional units or by the same operon. The correlation for subunit pairs encoded by the same operon is significantly higher than for those encoded by different transcriptional units (p = 0.002), as calculated by randomly shuffling the pairs between two groups of the same size 10^5^ times. (C) Comparison of protein abundance measurements for subunits from operon-encoded complexes versus other subunits from complexes encoded by different transcriptional units. Boxes represent quartile distributions, and whiskers extend up to 1.5× the interquartile range. The p value was calculated with Wilcoxon rank-sum test. Figure S1 shows these comparisons using protein abundance measurements combined from multiple organisms and with *E. coli* protein synthesis rates.

Many operons contain genes encoding different subunits of the same protein complex (Dandekar et al., 1998, Mushegian and Koonin, 1996) that can then be transcribed onto the same polycistronic mRNA. We first searched for heteromeric protein complexes of known structure from all prokaryotic organisms where at least two of the subunits are encoded by different genes from the same operon. In total, we identified 368 non-redundant pairs of subunits from the same heteromer encoded by different genes from the same operon (Figure 1A, left) from 70 different bacterial and archaeal species. This compares to 711 pairs encoded by different transcriptional units (*i.e.* translated from different mRNAs) from the same species (Figure 1A, right).

It has been suggested previously that a major advantage of operon-encoded complexes is their more efficient assembly because of smaller stochastic fluctuations in relative concentration than would occur if separate transcription steps were required for each subunit (Shieh et al., 2015, Sneppen et al., 2010, Swain, 2004). Unsurprisingly, it has been observed that proteins encoded by the same operon tend to be coexpressed (Wang et al., 2005). Similarly, in Figure 1B, we demonstrate a stronger correlation in *E. coli* protein abundance measurements (Wang et al., 2015) between pairs of subunits encoded by the same operon compared with pairs of subunits encoded by different transcriptional units.

If operons do provide a mechanism to minimize the stochasticity of assembly, then we can further predict that lowly expressed complexes should be more likely to be in operons because their assembly is inherently more stochastic (Kovács et al., 2009, Swain et al., 2002). This is supported by a highly significant (p = 6 × 10^−8^) tendency for operon-encoded subunits to be lower in abundance than subunits from complexes encoded by different transcriptional units (Figure 1C). Although there is an overlap between the groups, this suggests that lowly expressed genes encoding interacting subunits may have experienced stronger evolutionary pressure to be located on the same operon because of their more stochastic assembly. Alternatively, because of the efficiency of their assembly, operon-encoded subunits may only need to be expressed at lower levels.

### Operon-Encoded Subunits Tend to Be Encoded by Neighboring Genes and Form Large Interfaces

**Figure 2:**
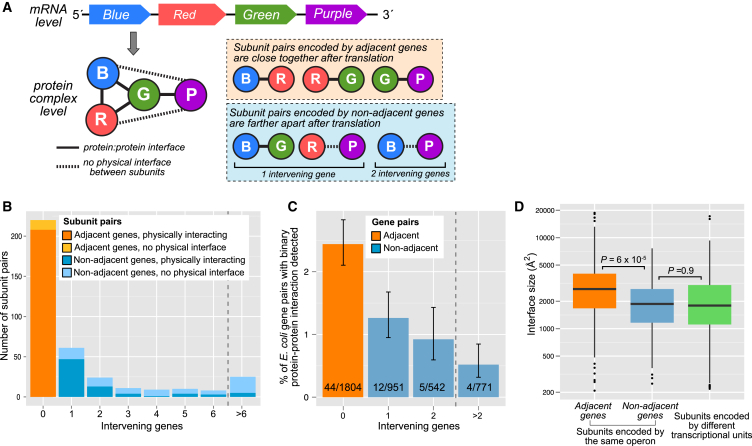
Genes Encoding Interacting Subunits of the Same Complex Tend to Be Close Together on an Operon (A) Illustration of how operon structure can be related to quaternary structure with a hypothetical four-subunit heteromer. Pairs of subunits from the same complex can be encoded by genes that are adjacent on an operon or farther apart. (B) Number of subunit pairs encoded by the same operon, grouped by the distance between their encoding genes. Subunit pairs are also divided into those that interact physically, which we define as forming an interface of >200 Å, and those that do not interact physically. (C) Percentage of pairs of *E. coli* genes from the same operon for which a binary yeast two-hybrid interaction could be detected. Error bars represent 68% Wilson binomial confidence intervals. (D) Distribution of interface sizes formed between physically interacting subunit pairs encoded by adjacent or non-adjacent genes on the same operon or between subunits encoded by different transcriptional units. Boxes represent quartile distributions, and whiskers extend up to 1.5× the interquartile range. The p values were calculated with Wilcoxon rank-sum test.

In addition to simply having genes encoding interacting subunits on the same operon, another way to enhance the efficiency of assembly would be to position the genes close together. If two genes encoding interacting subunits are close, then the newly translated subunits will also be close and more likely to encounter each other than if the two genes are farther apart (Figure 2A). In fact, the tendency for adjacent genes to code for interacting proteins has long been recognized (Dandekar et al., 1998, Mushegian and Koonin, 1996).

In Figure 2B, we plot the number of subunit pairs from the same complex by the distance between their genes within the operon. Strikingly, we see that 220 of 368 subunit pairs (59.8%) are encoded by adjacent genes. Furthermore, because not all subunit pairs from the same complex physically interact with each other (e.g., blue-purple and red-purple in Figure 2A), we note that the tendency to form a physical intersubunit interface within the complex is much higher between the adjacent (208 of 220) compared with non-adjacent (77 of 148) pairs (p = 5 × 10^−22^, Fisher’s exact test). Finally, this is supported further through analysis of a large set of *E. coli* binary protein-protein interactions (Rajagopala et al., 2014) where we confirmed that proteins encoded by adjacent genes are much more likely to interact (Figure 2C). Importantly, we show in Figure S2 that the tendency for interacting proteins to be close within an operon is highly significant compared with a null model in which gene order is randomized.

Figure 2D compares the sizes of interfaces formed between subunits encoded by adjacent genes, subunits encoded by non-adjacent genes from the same operon, and subunits encoded by different transcriptional units. We observe a highly significant tendency for adjacent subunits to be larger, although the interface size distribution is very broad and there is considerable overlap between the groups. This is especially interesting when considering that larger interfaces within a complex will usually assemble earlier than smaller interfaces (Levy et al., 2008, Marsh et al., 2013). This provides further evidence that operon structure appears to have been evolutionarily optimized for protein complex formation. Even when we consider only physically interacting proteins, those that form larger interfaces and are, therefore, likely to assemble earlier are much more likely to be encoded by adjacent genes.

The above observation could potentially have implications for our previous finding that evolutionary gene fusion events tend to conserve existing assembly pathways (Marsh et al., 2013) because fusion often occurs between adjacent genes. However, we show in Figure S3 that, even if only subunit pairs encoded by adjacent genes are considered, there still appears to be evolutionary selection for assembly-conserving fusions.

### Operon Gene Order Is Optimized for the Order of Protein Complex Assembly

The above results suggest that operon-encoded subunits will often be synthesized very close to each other within the cell. However, there is also a temporal component to this in that upstream genes will tend to be translated before downstream genes. This is first due to coupled transcription and translation, where the upstream gene that is transcribed first will also be translated first (Gowrishankar and Harinarayanan, 2004), and second to translational coupling, in which translating ribosomes can continue on to downstream genes (Oppenheim and Yanofsky, 1980). Therefore, if genes are arranged so that the gene order matches the order of subunit assembly, then the newly translated subunits will be more likely to interact quickly.

**Figure 3:**
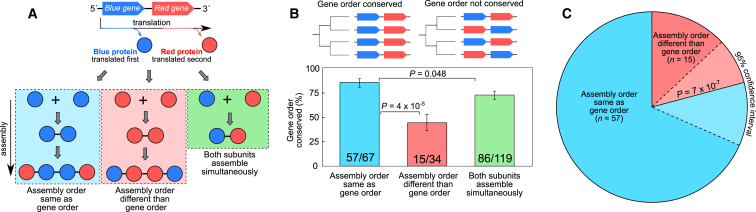
Operon Gene Order Reflects the Order of Protein Complex Assembly (A) Illustration of the three possible relationships between gene pair order and subunit assembly order. (B) Evolutionary conservation in pairs of adjacent genes encoding subunits of the same complex. The p values were calculated with Fisher’s exact test. Error bars represent 68% Wilson binomial confidence intervals. (C) When considering adjacent gene pairs with evolutionarily conserved gene order that encode different subunits of the same protein complex, the predicted assembly order is the same as the gene order in 57 of 72 cases. The p value was calculated with a binomial test.

We illustrate this in Figure 3A with the example of a hypothetical operon containing two adjacent genes, *blue* and *red*. If these genes encode different subunits of the same complex, then there are three possible relationships between gene order and assembly order. First, the assembly order could be the same as the gene order if the blue subunit that is translated first also assembles first. Second, the assembly order could be different than the gene order if the blue subunit assembles last. Finally, both subunits could assemble simultaneously, as would be the case for a simple heterodimer where the first step of assembly is the heteromeric interaction between different subunits.

Using our previous observation that assembly pathways can be predicted using interface sizes from three-dimensional structures of protein complexes (Marsh et al., 2013), here we predicted the assembly pathways for all operon-encoded heteromers in our dataset and classified each of the 220 adjacent gene pairs into one of these three groups. We then considered the tendency for gene order to be evolutionarily conserved in each group (Figure 3B). Interestingly, the evolutionary conservation of gene order is significantly higher in cases where it is the same as the predicted assembly order. This suggests that the evolutionary constraint on gene order is much stronger when it is optimized for assembly.

Next, we consider 72 gene pairs where gene order is evolutionarily conserved and where one subunit is predicted to assemble before the other. Figure 3C illustrates the striking correspondence between gene order and assembly order, with 57 pairs (79.2%) having the same assembly order as gene order (p = 7 × 10^−7^, binomial test). In contrast, when the gene order is not evolutionary conserved, only 10 of 29 gene pairs show correspondence between gene order and assembly order. Therefore, selection for ordered protein complex assembly appears to be a major evolutionary determinant of operon gene order.

We can also consider the relationship between gene order and assembly order for non-adjacent genes. Although the dataset is smaller, the relationship between gene order and assembly order appears to get weaker between genes that are more distant (Figure S4). This is likely due to weaker spatial and temporal coupling between non-adjacent genes that are translated farther apart from each other, as evidenced by the fact that subunits encoded by non-adjacent genes are much less likely to physically interact with each other (Figure 2B). Interestingly, the relationship between gene order and assembly order is stronger for proteins that interact physically, particularly those that form large interfaces. Similarly, subunit pairs encoded by adjacent genes where gene order and assembly order are the same tend to have significantly larger interfaces (Figure S4).

A possible alternative explanation for the correspondence between gene order and assembly order could be if earlier-assembling subunits need to be expressed at higher levels. Specifically, there is evidence of a linear relationship between expression levels and the proximity of genes to the start of operons (Lim et al., 2011, Nishizaki et al., 2007). This is weakly supported in the dataset used here, with proteins encoded by upstream genes showing a slight but not significant tendency to be more abundant (Table S1). Importantly, we find that protein expression levels show essentially no relationship with assembly and that gene order is a significantly better predictor of assembly order (Figure S5).

### Operon Gene Order Is Most Important for the Assembly of Lowly Expressed Proteins

Despite the strong correspondence between protein complex assembly and operon organization, there is still discordance between gene order and assembly order in >20% of cases where gene order is evolutionarily conserved. This suggests that there must be other factors besides assembly order that influence gene order conservation. For example, the operon order of enzyme genes is known to correlate with metabolic pathway order (Kovács et al., 2009, Zaslaver et al., 2006), although this seems unlikely to explain gene order in operon-encoded complexes. A search for gene ontology terms (Huntley et al., 2015) enriched in subunit pairs where gene order is either the same or different than assembly order revealed little that could account for the results observed here (Figure S6). Furthermore, if gene position can affect expression levels, as mentioned above, then there may be some evolutionary pressure to conserve gene order; for example, to not disrupt the relative subunit stoichiometry (Marsh et al., 2015).

The fact that operon gene order closely follows assembly order suggests that assembly must occur very shortly after protein synthesis because the more time newly synthesized subunits have to diffuse before assembly the less the order of gene expression should matter. Building on this, we hypothesize that the relationship between operon order and assembly order should be stronger for lowly expressed proteins. If they do not assemble quickly, diffusion will reduce the probability of two low-concentration subunits encountering each other. In contrast, the chance of interaction between highly expressed, abundant proteins will be greater, and so there is less need for assembly to occur close to the site of protein synthesis.

**Figure 4:**
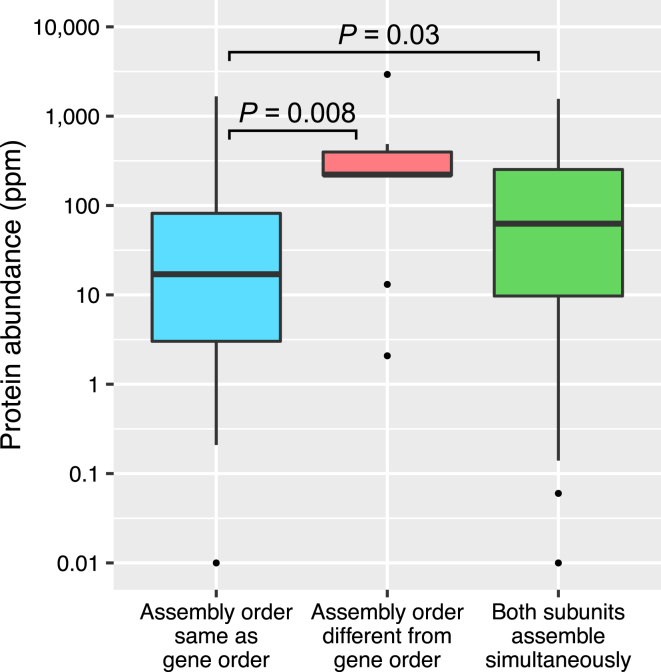
Cases Where Evolutionarily Conserved Gene Order Does Not Follow Assembly Order Tend to be Highly Expressed Boxes represent quartile distributions of protein abundance measurements, and whiskers extend up to 1.5× the interquartile range. The p values were calculated with a Wilcoxon rank-sum test. Figure S7 shows these comparisons using protein abundance measurements combined from multiple organisms and with *E. coli* protein synthesis rates.

In Figure 4, we plot the distributions of intracellular protein abundance measurements for subunits where conserved gene order follows assembly and for those where it does not. Those proteins where assembly order is the same as gene order tend to be much lower in abundance (p = 0.008, Wilcoxon test). Therefore, it appears that the correspondence between gene order and assembly order can mostly be attributed to lowly expressed proteins for which assembly is more stochastic. Interestingly, subunits where both assemble simultaneously are intermediate in abundance, consistent with the fact that gene order should show no correspondence with assembly in these cases.

## Discussion

Overall, a number of important conclusions can be drawn from these results. First, protein complex assembly within the cell appears to often follow the same ordered pathways that can be characterized experimentally and predicted computationally, at least in the case of operon-encoded complexes. Although there will certainly be some exceptions, particularly in cases where assembly chaperones are involved or subunits are translated in different parts of the cell, these results strongly support the physiological relevance of using in vitro or computational methods to study assembly.

Second, the remarkable correspondence between predicted assembly order and gene order further validates the utility of structure-based assembly predictions. Given the huge number of protein complex structures now known, this opens the door to future large-scale analyses of protein assembly pathways and their regulation, evolution, and role in biological function and disease.

This work also tells us something about where assembly occurs within the cell. For the low-abundance, operon-encoded complexes studied here, assembly must occur very close to the site of translation for gene order to have such a significant effect. In some cases, assembly may even occur co-translationally, involving at least one nascent chain still in the process of being translated (Duncan and Mata, 2011, Wells et al., 2015), as has been demonstrated recently for the operon-encoded bacterial luciferase complex (Shieh et al., 2015).

Finally, these results strongly support the biological importance of assembly pathways and suggest that co-ordinating both the timing and location of translation is important for maximizing the efficiency of stochastic protein complex assembly. The fact that operon gene order has been optimized for assembly order in many protein complexes suggests that assembly order is often very important and that there is significant benefit from tightly co-ordinating gene expression and protein assembly. Given that eukaryotes do not have operons that allow multiple protein subunits to be translated from the same polycistronic mRNA, it will be interesting to systematically investigate which other mechanisms might be employed to enhance the efficiency of assembly.

## Experimental Procedures

### Protein Structural Datasets

We started with the full set of prokaryotic X-ray and electron microscopy structures in the PDB on June 12, 2014. We considered all heteromeric pairs of subunits from the same complex, defined as having at least two different protein chains of ≥30 residues each and mapping to different UniProt sequences from a single species. Complexes with known quaternary structure assignment errors (Levy, 2007) were excluded. Very large complexes with >24 subunits were excluded, because we have not shown that the assembly of these can be predicted accurately from their structures. Heteromeric subunit pairs were filtered for redundancy at the level of 50% sequence identity.

### Mapping Subunit Pairs to Operons

Operon datasets were downloaded from the DOOR^2^ database (Mao et al., 2014). Relevant datasets were identified based on the species and strain of each gene pair. After converting GI numbers to UniProt accession identifiers in each dataset, the set of gene pairs was mapped to the operon data. Operons encoding both members of a pair were added to a reference dictionary, with the locus and directionality of each gene being used to arrange constituent genes in order of expression. In rare cases where the copy number of a gene within an operon was found to be greater than one, the position of the gene in the operon was taken to be that of the first copy to be encountered, reading in the 5′ to 3′ direction. The set was then filtered to remove redundant operons (i.e., identical operons from similar strains or species). In total, 368 gene pairs (220 adjacent) were mapped to 192 unique operons, with the remaining 711 pairs being expressed in different transcriptional units. These are provided in Dataset S1. Similarly, we also mapped a set of 2,562 binary protein-protein interactions (IM-22059) (Rajagopala et al., 2014) to the *E. coli* K-12 W3110 operons to calculate the result in Figure 2C (provided in Dataset S2).

To assess whether the gene order of a pair was evolutionary conserved, we used the STRING v9.1 database (Franceschini et al., 2013). For each pair, we manually assessed, using the STRING online interface, whether all occurrences of a given gene pair shared the same gene order within their local evolutionary group as defined in STRING. This is at the level of phylum (*e.g.* Firmicutes or Euryarchaeota) or class for proteobacteria, with all groups provided in Dataset S1. Gene pairs present across only a very limited evolutionary range (less than three genera) were not considered to be evolutionarily conserved. Gene pairs associated with evolutionary gene fusion events were identified as those sharing >40% sequence identity with a gene pair with evidence for fusion in STRING, similar to what has been done previously (Marsh et al., 2013).

### Abundance Measurements

We mapped all protein complex subunits in our dataset against the sequences of prokaryotic proteins from PaxDB v4.0 (Wang et al., 2015), selecting abundance measurements with >90% sequence identity to a subunit. The results in Figures 1 and 4 only use abundance measurements from *E. coli*, but the analyses in the Figures S1, S5, and S7 and Table S1 are repeated using combined measurements from all available prokaryotes and also using protein synthesis rates derived from ribosomal profiling (Li et al., 2014).

### Prediction of Assembly Pathways

Ordered protein complex assembly pathways were predicted in a manner very similar to what has been done previously (Marsh et al., 2013). First, the complex is considered in terms of its constituent subunits and the sizes of the interfaces that can be formed between any pair of subunits are calculated with AREAIMOL (Winn et al., 2011). Our model assumes that assembly will proceed via formation of the largest possible interface. The process is then repeated by calculating all possible interfaces that could form between subunits and subcomplexes until the full complex is assembled. To define which of a pair of subunits assembles first and which assembles later, we consider the first step of assembly that brings the two subunits together within the same (sub)complex. Whichever subunit was part of a larger subcomplex prior to this step is defined as assembling first. For example, in the blue pathway in Figure 3A, the blue subunit homodimerizes first and then interacts sequentially with the free red subunits, so the blue subunit is defined as assembling first. If, alternatively, the first step of assembly had been a heterodimerization between the blue and red subunits, then both subunits would be classified as assembling simultaneously. The relative order of assembly for each subunit pair is included in Dataset S1, and all predicted assembly pathways are provided in Dataset S3. The source code for predicting assembly pathways from protein complex structures is available at http://github.com/marshlab/assembly-prediction.

The full set of gene ontology associations for complexes where assembly order and gene order are the same or different is provided in Dataset S4.

## Author Contributions

J.M. conceived and designed the research. J.W., T.B., and J.M. performed the computational analyses. J.M. wrote the manuscript with contributions from all authors.

## Supplemental Information

Document S1. Figures S1–S7 and Table S1

Dataset S1. List of All Gene/Subunit Pairs Used in This Study and Their Relevant Properties, Related to Figure 1

Dataset S2. Pairs of *E. coli* Genes from the Same Operon with or without Evidence of Binary Protein-Protein Interaction, Related to Figure 2

Dataset S3. Predicted Assembly Pathways for Heteromeric Protein Complexes, Related to Figure 3

Dataset S4. Enrichment of Gene Ontology Terms Associated with Protein Complex Subunits Where Assembly Is Either the Same or Different Than Operon Gene Order, Related to Figure 3

Document S2. Article plus Supplemental Information
