# Supplementary material for: Operon Gene Order Is Optimized for Ordered Protein Complex Assembly
Source: Cell Rep. 2016 Jan 21;14(4):679–85. doi: 10.1016/j.celrep.2015.12.085 (PMC4742563; doi:10.1016/j.celrep.2015.12.085)
Supplement: Document S1. Figures S1–S7 and Table S1 [file mmc1.pdf]

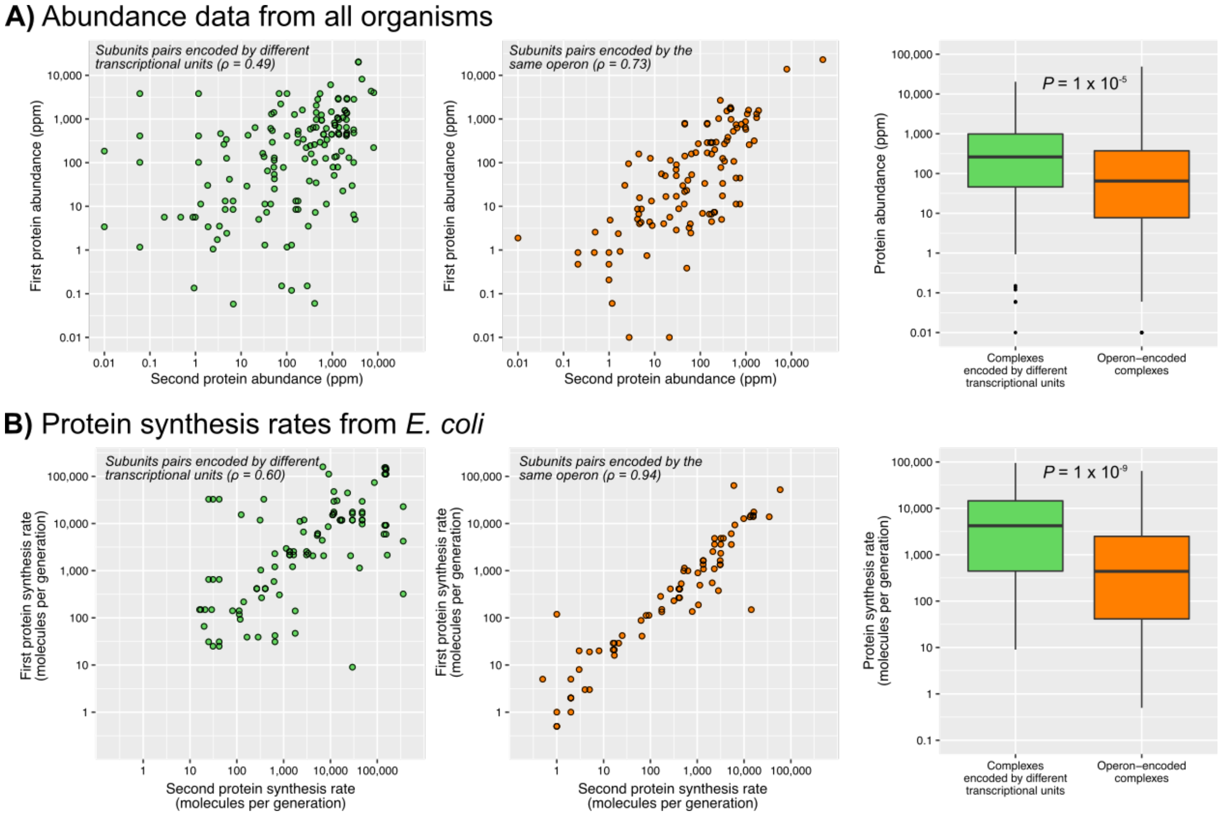

Figure S1: Comparison of subunit pairs encoded by different transcriptional units vs. those encoded by the same operon, using abundance measurements combined from multiple organisms, or absolute protein synthesis rates from *E. coli*, related to Figure 1*.* A) Same as Fig. 1B-C, except using PaxDB abundance measurements from all prokaryotes instead of just *E. coli*. **B)** Same as Fig. 1B-C, except using *E. coli* absolute protein synthesis rates derived from ribosomal profiling experiments (Li et al., 2014). The correlations for subunit pairs encoded by the same operon are significantly higher than for those encoded by different transcriptional units in both datasets (P = 0.004 for A and $<10^{-5}$ for B), calculated by randomly shuffling the pairs between two groups of the same size 10^5 times.

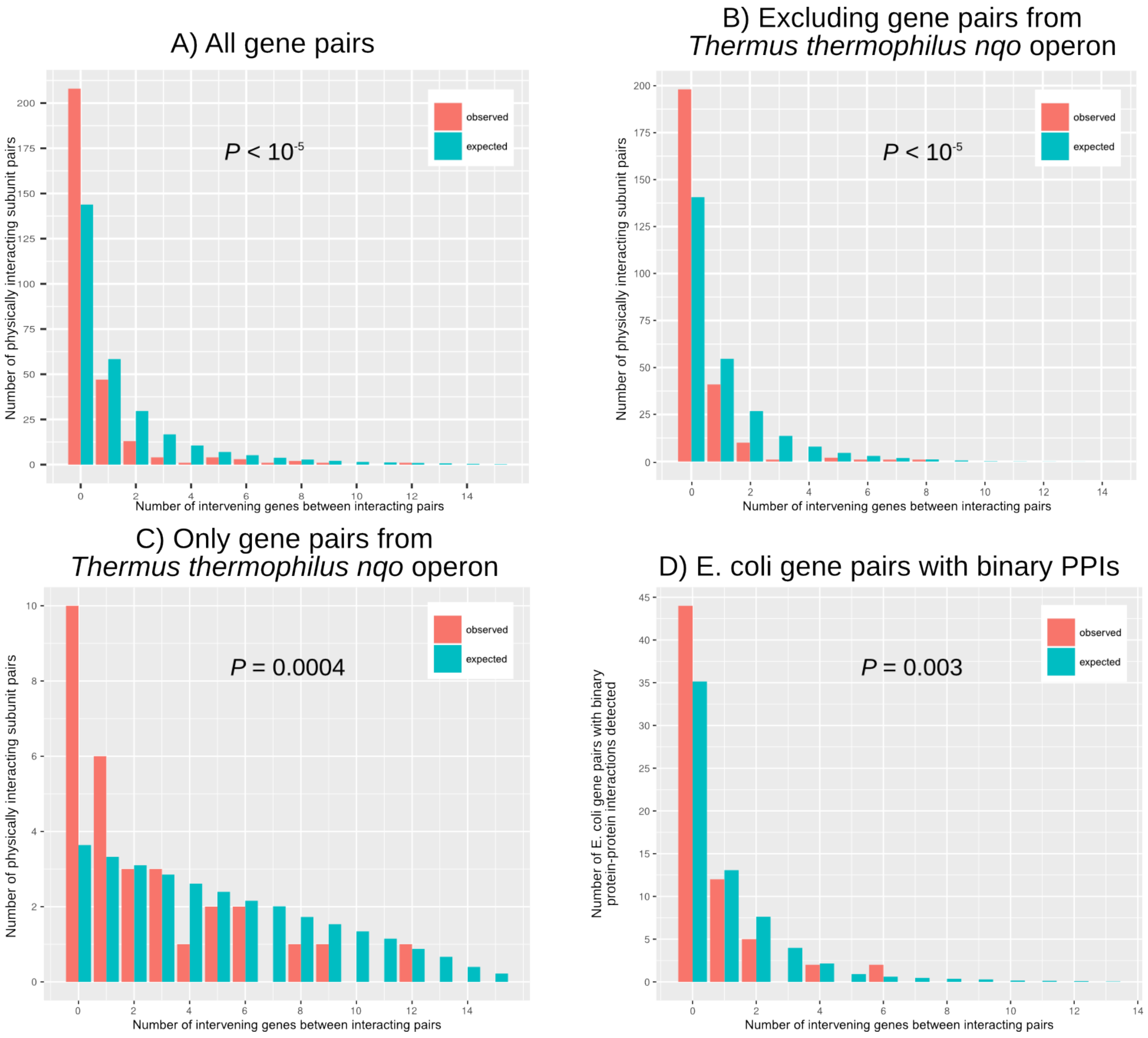

Figure S2: Relationship between operon gene proximity and tendency to physically interact, related to Figure 2. These plots show the number of physically interacting protein pairs (intersubunit interface >200 Å) observed (red), compared to the number expected if the gene order of the operon is randomly shuffled (turquoise). Panel **A)** shows the comparison with all operon gene pairs from our main dataset (as in Fig. 2B), and **D)** shows the *E. coli* pairs with binary protein-protein interactions (as in Fig. 2C). More than half (78/148) of the non-adjacent gene pairs come from a single operon: the *nqo* operon from *Thermus thermophilus* encoding complex I (operon #116617 in Dataset S1). No other operon contributes more than 6 gene pairs. Thus we also present these analyses excluding gene pairs from the *nqo* operon in **B)** and only including *nqo* in **C)**. For each plot, the operons were shuffled 10^5 times, and the *P*-values represent the probability of seeing ≤ the total number of intervening genes between interacting proteins.

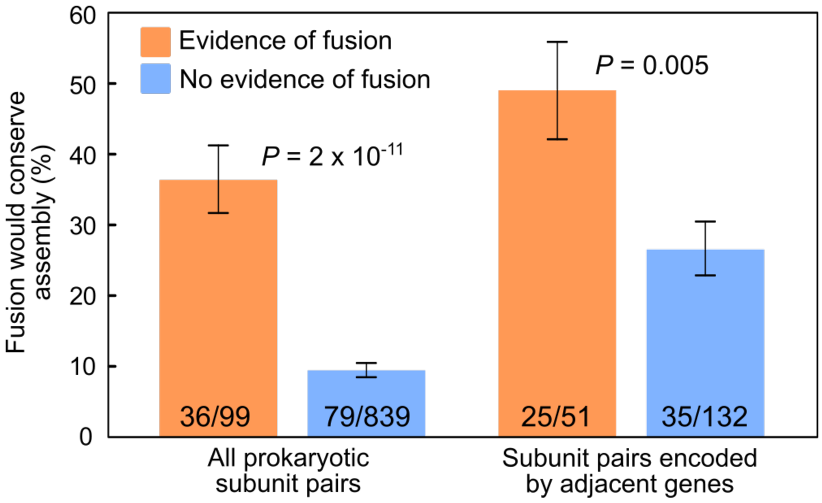

Figure S3: Evolutionary conservation of protein complex assembly pathways by gene fusion events, related to Figure 2. Percentage of cases in which a covalent fusion between protein complex subunits would conserve existing assembly pathways for subunit pairs from heteromers with >2 total subunits, where there is evidence of an evolutionary fusion in some other species (orange), and those where there is no evidence of fusion (blue). On the left, all non-redundant subunit pairs from our dataset are considered, whereas on the right, only subunit pairs encoded by adjacent genes from the same operon are included. *P*-values are calculated using Fisher's exact test. Error bars represent 68% Wilson binomial confidence intervals.

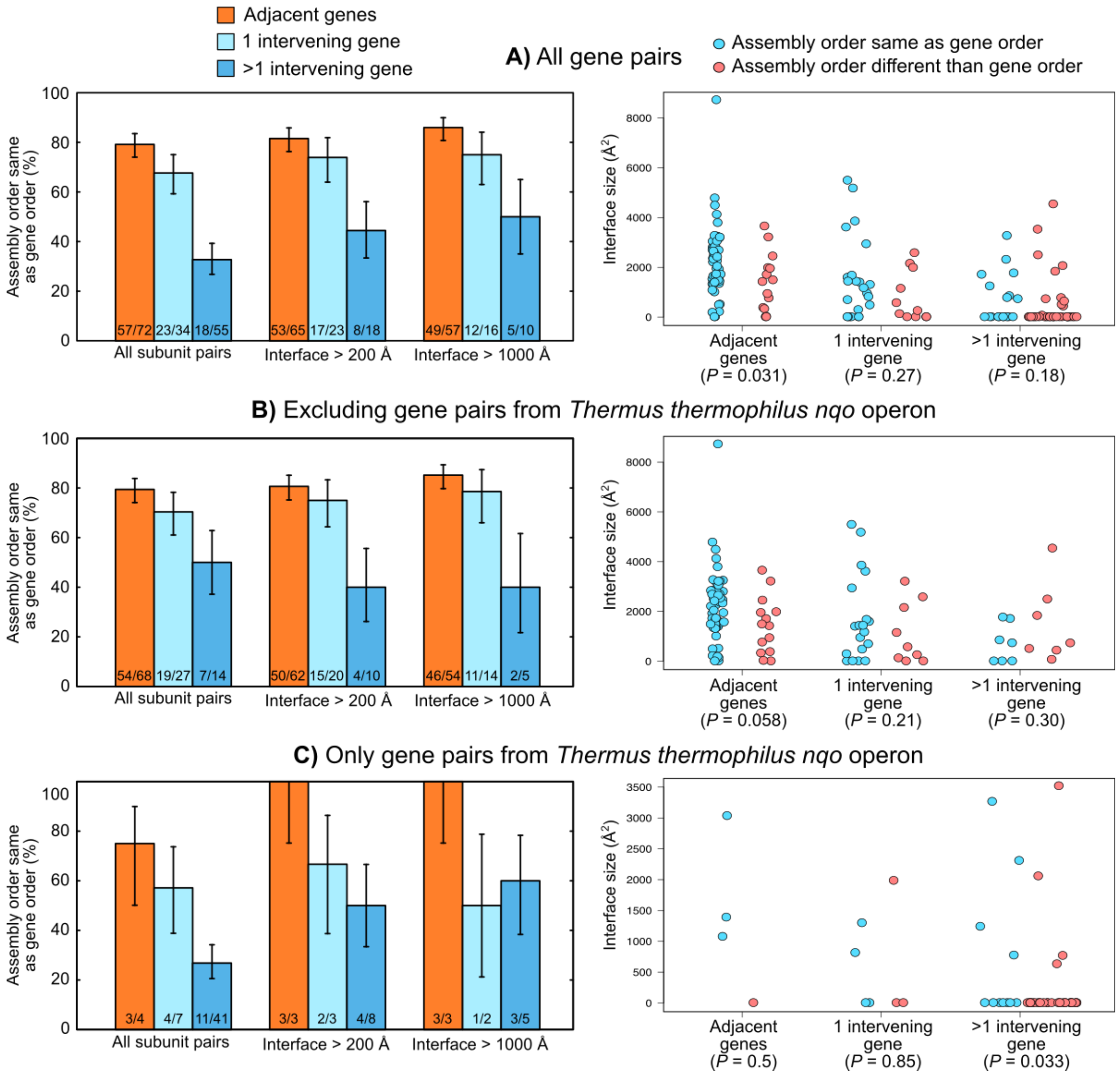

Figure S4: Comparison of the relationship between gene order, assembly order and interface size for adjacent and non-adjacent gene pairs, related to Figure 3. Plots on the left side show the % of gene pairs where evolutionarily conserved gene order is the same as assembly order. They are split into adjacent gene pairs, gene pairs with a single intervening gene, and gene pairs with multiple intervening genes. Error bars represent 68% Wilson binomial confidence intervals. Plots on the right side show a comparison between interface sizes for subunit pairs where assembly order is either the same as gene order or different. *P*-values are calculated with the Wilcoxon rank-sum test. Since nearly half of the non-adjacent gene pairs come from the *T. thermophilus nqo* operon, as mentioned in Fig. S2, we also show these comparisons excluding pairs from this operon, or only including these genes.

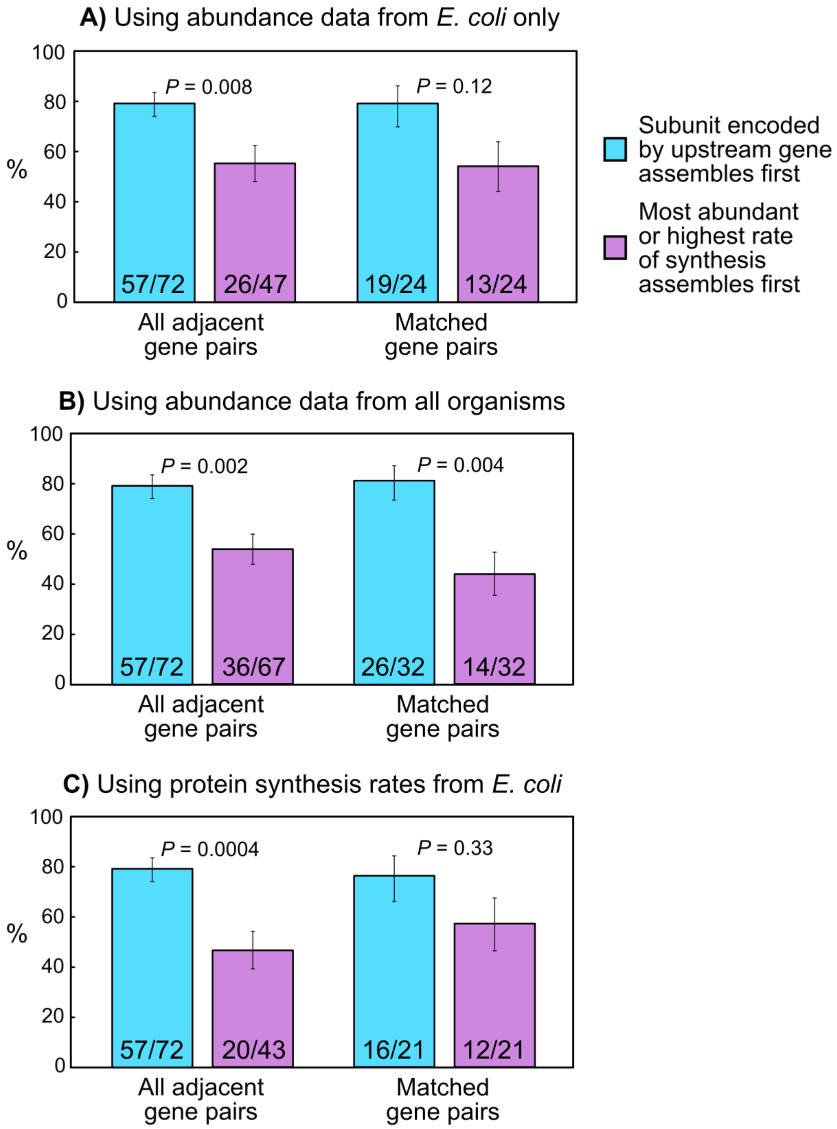

Figure S5: Gene order is a stronger predictor of assembly order than protein expression levels, related to Figure 3. Due to the previous observations that upstream genes in operons tend to be more highly expressed, we tested whether this could possibly affect our conclusion that gene order is optimized for assembly order. We tested whether upstream subunits are more likely to assemble first compared to subunits that are more abundant or have a higher rate of synthesis. *"All adjacent gene pairs"* includes all evolutionarily conserved gene pairs with abundance or synthesis measurements available for both proteins, whereas *"Matched gene pairs"* only includes the much smaller subsets of pairs where abundance measurements are available for both and one subunit is predicted to assemble before the other, so that the same pairs are used for each comparison. Comparisons were performed using **(A)** abundance data from *E. coli* only, **(B)** abundance data from all organisms, and **(C)** absolute protein synthesis rates from *E. coli*. Error bars represent 68% Wilson binomial confidence intervals. *P*-values are calculated with Fisher's exact test.

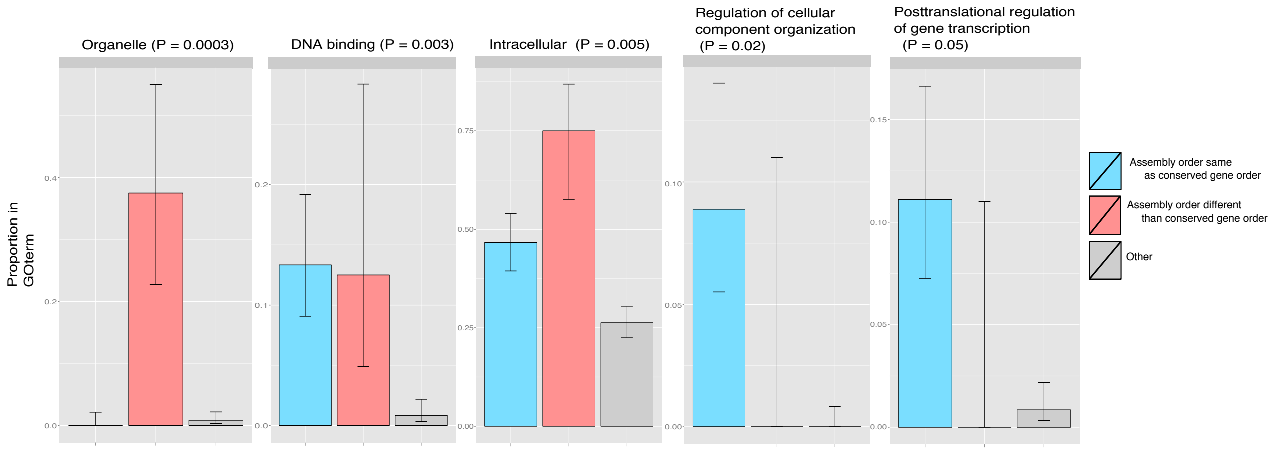

Figure S6: Enrichment of Gene Ontology terms, related to Figure 3. Three groups of complexes are considered in terms of their functional annotations: assembly order same as conserved gene order (blue), assembly order different than conserved gene order (red) and other (grey). The other group includes the complexes where the gene order is not conserved and/or where there is no ordered assembly. The significance of the distribution of each GO term with respect to the three groups was calculated with Fisher's exact test, using *P*-values simulated with 2×10^6 Monte Carlo iterations. The GO terms were then filtered for redundancy: if two terms co-occur in >50% of all proteins in the GOA database where either of the two terms are observed, then only the term with the lower *P*-value was considered in the non-redundant set. Error bars represent 68% Wilson binomial confidence intervals. The top five non-redundant GO terms with the lowest *P*-values are shown here, with results for all GO terms given in Dataset S4. Although the enrichment of "organelle" for complexes where assembly order is different than gene order appears striking, it is based upon only three complexes covering quite disparate activities (ribosomal L7/12 stalk, topoisomerase VI and CheY:CheZ), so it is difficult to ascribe much biological meaning to this.

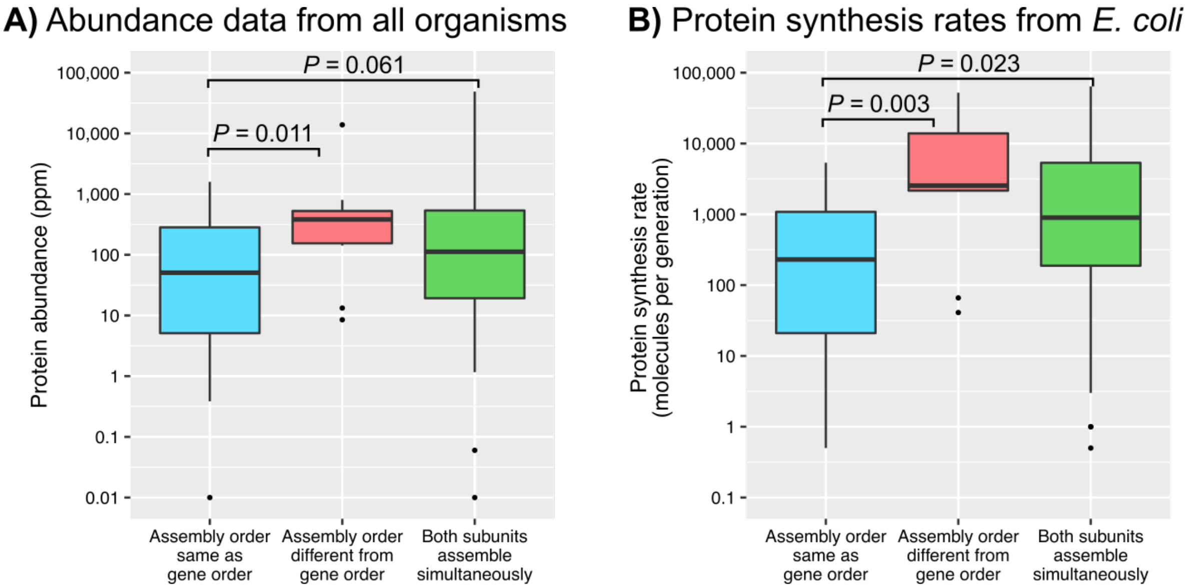

Figure S7: Same comparison as in Figure 4 using abundance measurements from all organisms or absolute protein synthesis rates from *E. coli*, related to Figure 4.

Table S1: Relationship between protein abundance and gene order for adjacent genes encoding different subunits of the same heteromeric complex, related to Figure 3. Overall, there may be a slight tendency for proteins encoded by upstream genes to be more abundant, although none of these results are close to statistically significant with the binomial test. In addition, when considering rate of protein synthesis derived from ribosomal profiling (Li et al, 2014), the upstream genes actually show lower rates of synthesis, although this is also not significant.

	**Subunit encoded by upstream gene is more abundant (*E. coli* only)**	**Subunit encoded by upstream gene is more abundant (All species)**	**Subunit encoded by upstream gene has a higher rate of protein synthesis**
**Gene order is evolutionarily conserved**	26/47 (55.3%)	36/67 (53.7%)	20/43 (46.5%)
**Gene order is not evolutionarily conserved**	5/10 (50.0%)	10/16 (62.5%)	3/9 (33.3%)
**Total**	31/57 (54.5%)	46/83 (55.4%)	23/42 (44.2%)

Dataset S1: List of all gene/subunit pairs used in this study and their relevant properties, related to Figure 1.

Dataset S2: Pairs of *E.* coli genes from the same operon with or without evidence of binary protein-protein interaction, related to Figure 2.

Dataset S3: Predicted assembly pathways for heteromeric protein complexes, related to Figure 3.

Dataset S4: Enrichment of Gene Ontology terms associated with protein complex subunits where assembly is either the same or different than operon gene order, related to Figure 3.
